# Semi-Synthesis and Analysis of Chemically Modified Zif268 Zinc-Finger Domains

**DOI:** 10.1002/open.201100002

**Published:** 2012-01-02

**Authors:** Friederike Fehr, André Nadler, Florian Brodhun, Ivo Feussner, Ulf Diederichsen

**Affiliations:** [a] Georg-August-Universität Göttingen, Institut für Organische und Biomolekulare ChemieTammannstrasse 2, 37077 Göttingen (Germany), Fax: (+49)-551-39-2944 E-mail: udieder@gwdg.de; [b] Georg-August-Universität Göttingen, Albrecht-von-Haller Institut für PflanzenwissenschaftenJustus-von-Liebig-Weg 11, 37077 Göttingen (Germany)

**Keywords:** expressed protein ligation, metalloproteins, peptide synthesis, proteins, zinc-finger domains

## Abstract

Total synthesis of proteins can be challenging despite assembling techniques, such as native chemical ligation (NCL) and expressed protein ligation (EPL). Especially, the combination of recombinant protein expression and chemically addressable solid-phase peptide synthesis (SPPS) is well suited for the redesign of native protein structures. Incorporation of analytical probes and artificial amino acids into full-length natural protein domains, such as the sequence-specific DNA binding zinc-finger motifs, are of interest combining selective DNA recognition and artificial function. The semi-synthesis of the natural 90 amino acid long sequence of the zinc-finger domain of Zif268 is described including various chemically modified constructs. Our approach offers the possibility to exchange any amino acid within the third zinc finger. The realized modifications of the natural sequence include point mutations, attachment of a fluorophore, and the exchange of amino acids at different positions in the zinc finger by artificial amino acids to create additional metal binding sites. The individual constructs were analyzed by circular dichroism (CD) spectroscopy with respect to the integrity of the zinc-finger fold and DNA binding.

## Introduction

The understanding of gene-regulation mechanisms aiming for early identification of morbid cells or false DNA sequences is one main focus in current postgenomic research towards medicinally relevant problems. Selective gene expression is mainly accomplished by the interaction of DNA with selective binding proteins containing specific DNA binding domains, such as helix-turn-helix (HTH) and zinc-finger motifs.^1^ It is assumed that in higher eukaryotes more than 15 000 zinc-finger motifs occur in 1000 proteins, which makes it one of the most abundant motifs.^2^ Zinc-finger motifs possess the ability to recognize DNA sequences selectively and are part of most transcription factors.^3^ They contain a zinc ion in tetrahedral coordination with cysteine and histidine residues, resulting in the eponymous simple ββα fold.^4^ Zinc-finger motifs are subdivided into classes with respect to the amino acid residues coordinating the structural zinc.^5^ The best investigated zinc-finger domain of the canonical Cys 2 His 2 type belongs to the murine transcription factor Zif268 and serves as a useful model to analyze protein–DNA interactions.^6^ Since Zn^2+^ displays different properties within a wide range of proteins, its functions are classified into catalytically active, with a readily exchangeable water ligand at the coordination site and structure determining, where all coordination sites are occupied by donor atoms of the protein side chains.^7^ In zinc fingers, the metal contributes only to the ββα fold and plays no catalytic or functional role. In Zif268, the DNA binding motif is composed of a tandem repeat of three zinc fingers, each consisting of about 30 amino acids, serving as recognition units and binding to the major groove of B-DNA. Each of the three zinc fingers has the ability to recognize a sequence of three to four nucleobases through four conserved amino acids in the α-helical part and is linked by a short well-conserved sequence to the following one. It is known that at least two zinc-finger units are required for selectivity.^7^, ^8^

Aim of the current work was the incorporation of an additional metal binding site into the zinc-finger motif allowing functionalization of a sequence-specific DNA binder placed in the major groove. A functional metal binding site placed in close proximity to the structural metal center might serve as spectroscopic probe or allows for cooperative effects with respect to catalytic activity.^9^ In particular, metal-based nucleases often contain two metal ions, such as Mg^2+^, Mn,^2+^ or Zn^2+^, in close proximity that are involved in the cleavage of the DNA phosphodiester backbone.^10^ Despite numerous investigations on native metalloproteins and their functions to understand fundamental principles in chemistry and biology,^11^ little is known about strategies accomplishing artificial metal-containing, biologically active peptides or proteins.^12^ In this respect, the redesign of native proteins has an advantage over the de novo design of peptides, as it proceeds from a known scaffold requiring only minor modulation.^7^, ^13^ Zinc-finger motifs are also convenient from the synthetic aspect, because cysteine residues in a tandem repeat of a conserved amino acid sequence offer potential native chemical ligation sites in attractive intervals. Furthermore, by coordinating a metal ion, zinc fingers adopt a defined peptide fold. The semi-synthesis and characterization of the zinc-finger domain of the Zif268 transcription factor by expressed protein ligation (EPL) is reported. Besides the native binding domain, modifications within the sequence of the third zinc finger are investigated, such as single amino acid mutations, attached fluorophore and constructs with artificial metal-chelating amino acids.9b

## Results and Discussion

### Strategy

The zinc-finger domain of the transcription factor Zif268 (Egr1) is a well-known and characterized motif, and for these reasons, was chosen as selective DNA binding motif. The sequence contains 90 amino acids and is hardly accessible by standard fluorenylmethyloxycarbonyl (Fmoc) solid-phase peptide synthesis (SPPS). A semi-synthetic approach based on EPL was applied to assemble the full-length peptide. Thereby, the first two zinc fingers (Zf12, amino acids 1–64) were expressed in *Escherichia coli*, whereas the third zinc finger (Zf3, amino acids 65–90) was synthesized by Fmoc SPPS. Native chemical ligation (NCL) yielded the desired sequence of 90 amino acids.

### Generation and purification of Zf12

**Figure 1:**
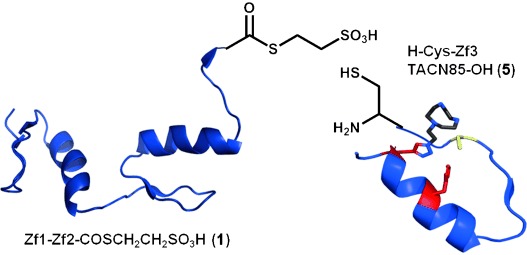
Image of the expressed C-terminal peptide thioester Zf12 (amino acids 1–64, left) and the peptide Zf3 (amino acids 65–91, right) with an N-terminal cysteine and a modification with an additional metal binding site at His 85 (modified Protein Data Bank (PDB) entry: IAAY^6^).

**Figure 2:**
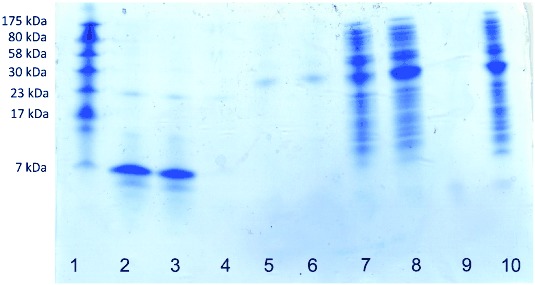
Tris-tricine SDS-PAGE (10–20 %) of Zf12 (8 kDa); lanes: 1=molecular weight standard; 2=fraction 2 (Zf12); 3=fraction 1 (Zf12); 4=cleavage; 5=flow through (FT) 3; 6=FT 2; 7=FT 1; 8=lysate; 9=inclusion bodies; 10=pellet.

After amplification of the open reading frame of the zinc-finger domain (Zf12) by polymerase chain reaction (PCR), the desired DNA sequence was cloned in-frame into the pTXB1 expression vector. The Zf12 peptide was expressed in *E. coli* in fusion with a mini-intein (*GyrA* gene of *Mycobacterium xenopi*) and a chitin binding domain (CBD) from *Bacillus circulans*.^14^ After affinity chromatography using immobilized chitin beads, the Zf12 peptide was cleaved from the resin by thioesterification adding the sodium salt of 2-mercaptoethanesulfonic acid (MESNa; Figure 1). The thioester peptide **1** (Zf12) was eluted, and further purified by reverse phase (RP)-HPLC giving a final yield of 5 mg L^−1^ cell culture. From tris-tricine-based sodium dodecyl sulfate polyacrylamide gel electrophoresis (SDS-PAGE), it was concluded that thioester **1** was eluted from the affinity column within the first fractions (Figure 2);^15^ the constitutional integrity of peptide **1** was confirmed by HRMS-ESI.

#### SPPS of Zf3 and Zf3 modifications

**Figure 3:**
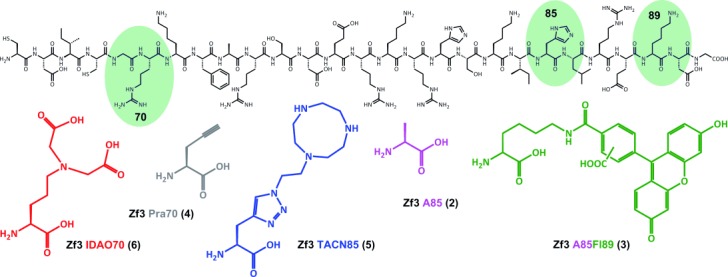
Zinc-finger 3 (Zf3) fragment prepared by SPPS. Exchanged amino acids Arg 70, His 85 and Lys 89 are indicated together with newly introduced amino acids alanine (Zf3 A85, 2), alanine and 5(6)-carboxyfluoresceine (Zf3 A85 L89, 3), propargylglycine (Zf3 Pra70, 4), triazole-linked triazacyclononane (Zf3 TACN85, 5), and iminodiacetic acid ornithine (Zf3 IDAO70, 6).^12^

The syntheses of Zf3 and its variants were achieved by a combination of manual and automated standard microwave-assisted Fmoc-based SPPS on Wang resin. Hydroxybenzotriazole (HOBt) and *O*-benzotriazole-*N,N,N′,N*′-tetramethyl-uronium-hexafluoro-phosphate (HBTU) were used as coupling reagents. Besides the natural sequence of Zf3, five variants were synthesized. Modification Zf3 A85 (**2**) contains histidine at position 85 exchanged for an alanine (Figure 3). His 85 is one out of four ligands coordinating the structural zinc ion in the system. Mutation to alanine creates a vacant coordination site at the metal center offering the potential for catalytic activity.^16^ This variant was further modified by introduction of a 5(6)-carboxyfluoresceine fluorophore (Zf3 A85L89, **3**) to the second last C-terminal amino acid Lys 89. Therefore, peptide **3** lacks His 85 and contains 5(6)-carboxyfluoresceine coupled to the lysine side chain. During SPPS, the lysine side chain was allyloxycarbonyl (alloc)-protected, and coupling to the fluorophore was activated by HOBt, *N*,*N*′-diisopropylcarbodiimide (DIC), and *N*-ethyldiisopropylamine (DIPEA). By using the zinc finger containing a fluorophore, zinc-finger ligation could be monitored by UV, and this modified zinc finger will also be valuable for following its interactions with DNA by Förster resonance energy transfer (FRET).^17^

In variant Zf3 Pra70 (**4**), the arginine residue at position 70 was exchanged with the artificial amino acid propargylglycine (Pra). This amino acid with its acetylene side chain permits the introduction of various functionalities into the protein by [2+3] cycloaddition with azides.^18^ Azide-functionalized moieties, such as fluorophores or metal chelators, can thereby be introduced at position 70 of the zinc finger. Native Arg 70 maintains a phosphate contact to the DNA backbone, which offers the possibility to introduce a broad range of functional groups close to a potential DNA cleavage site.^6^ Furthermore, with Zf3 TACN85 (**5**), a triazole was introduced as an imidazole mimic of the metal-chelating amino acid His 85 alkylated by a 1,4,7-triazacyclononane (TACN) group to create a second metal binding site. Preparation of Fmoc–propargylglycine—OH by [2+3] cycloaddition as well as an azide-functionalized *N,N*-bis-Boc-protected TACN was reported previously.9b The appropriate Fmoc–TACN—OH building block was incorporated in the peptide by SPPS under standard HOBt/HBTU conditions. The triazole in Zf3 TACN85 (**5**) mimics the histidine and additionally creates a second metal binding site in close proximity to the natural one.^19^

The zinc-finger variant Zf3 IDAO70 (**6**) contains an ornithine analogue, iminodiacetic acid ornithine (IDAO), which is functionalized with two acetic acid groups at its amine side chain, as an artificial metal-chelating amino acid.9b Arg 70 was exchanged with the Fmoc–IDAO—OH building block that was manually coupled, protected as bis-*tert*-butylester under 1-hydroxy-7-azabenzotriazole (HOAt)/HATU coupling conditions in threefold excess by using a microwave-assisted protocol.

### Native chemical ligation

**Figure 4:**
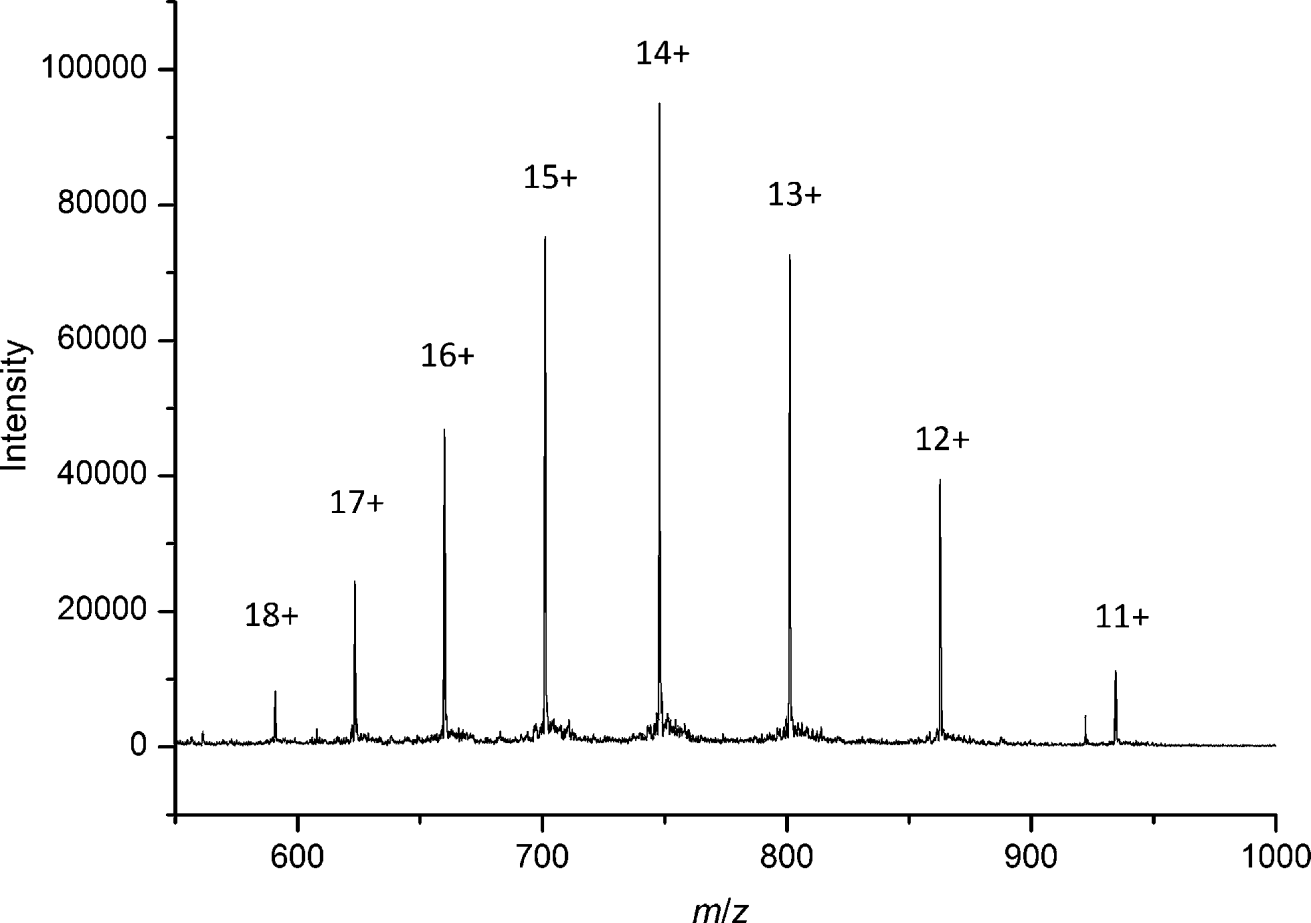
ESI-MS spectrum of the ligation product Zf13 A85 L89 (3).

The full-length zinc-finger proteins were generated by native chemical ligation of the expressed Zf12 thioester peptide **1** and the respective synthetic Zf3 peptide with an N-terminal cysteine residue.^20^ These unfolded zinc fingers easily form disulfide bridges, and reduced cysteines are crucial for the ligation step. Therefore, the peptides were treated with the tris(2-carboxyethyl)phosphine (TCEP), as a reducing agent, carefully balancing the pH. Because reduction with TCEP only occurs at a pH of about 4, and the optimal pH for NCL is in the range of 7.8 to 8, Zf3 peptides were dissolved in a millimolar range (threefold excess with respect to Zf12) under argon in 10 mm phosphate buffer (pH 4) containing 20 mm TCEP and 6 m guanidinium hydrochloride (Gnd**⋅**HCl) (1 *v*/*v*). The reduction was complete after 2 h, as confirmed by ESI-MS. The expressed Zf12 was solved in a millimolar range in 500 mm phosphate buffer and 6 m Gnd**⋅**HCl (1.2 *v*/*v*) at pH 8. The two solutions were mixed subsequently, and the resulting pH was kept between 7.8 and 8. Conversion into the ligation products was followed by analytical RP-HPLC. The respective Zf13 ligation product was obtained after 6–12 h, purified by RP-HPLC, and subsequently analyzed by ESI-MS. Because disulfide formation was indicated by ESI-MS, reduction was performed at pH 4 by reaction with 20 mm of TCEP under argon for 2 h. The pH was then adjusted to 8 to avoid amine protonation and allow Zn^2+^ coordination. As an example, the ESI-MS spectrum for the reduced modified zinc-finger construct Zf13 A85L89 (**3**) is presented in Figure 4.

### Structural analysis and DNA interaction of Zf13 constructs

**Figure 5:**
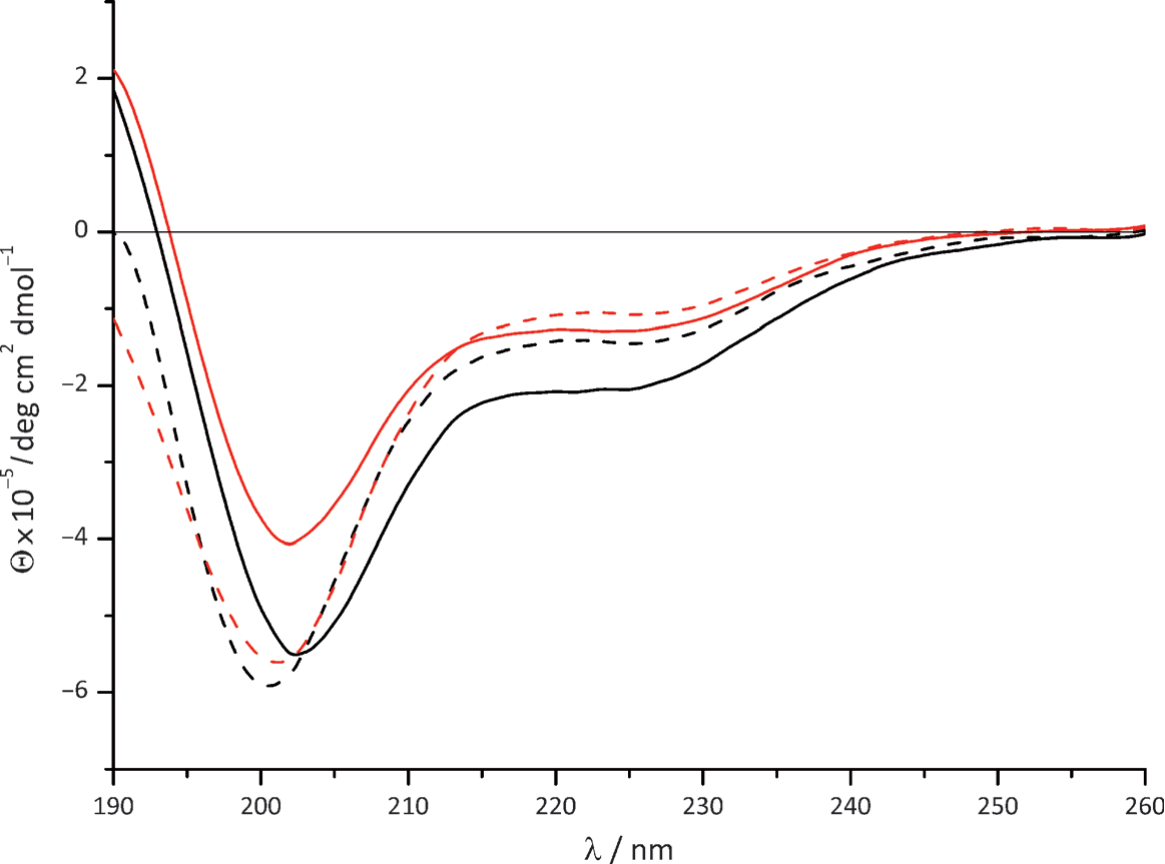
CD spectra of native Zf3 (17 μm;black) and modified Zf3 TACN85 (26 μm;red) in the absence (– – –) and presence (—) of 65 μm ZnSO_4_.

CD measurements were used to study the influence of the modifications on the structure of Zf3 peptides. The zinc-induced protein folding can be monitored by CD spectroscopy, mainly by following a hypsochromic shift of the negative maximum close to 200 nm.^21^ All reduced Zf3 analogues were analyzed in absence and presence of ZnSO_4_. The reported blueshift was observed for all analogues upon addition of an excess Zn^2+^; exemplarily, in Figure 5 spectra for native peptide Zf3 and the analogue containing an additional metal binding site, Zf3 TACN85 (**5**) are shown. In Zf3 TACN85 (**5**), the metal-coordinating His 85 is replaced by a triazole that is also likely to be involved in zinc coordination, and as such, the CD spectra of Zf3 and Zf3 TACN85 (**5**) are quite similar. Also, mutant Zf3 A85 (**2**), lacking the histidine metal coordination site is still able to provide the correct fold (data not shown), indicating that the typically ββα zinc-finger fold is not significantly affected by the Zf3 mutations.16a

**Figure 6:**
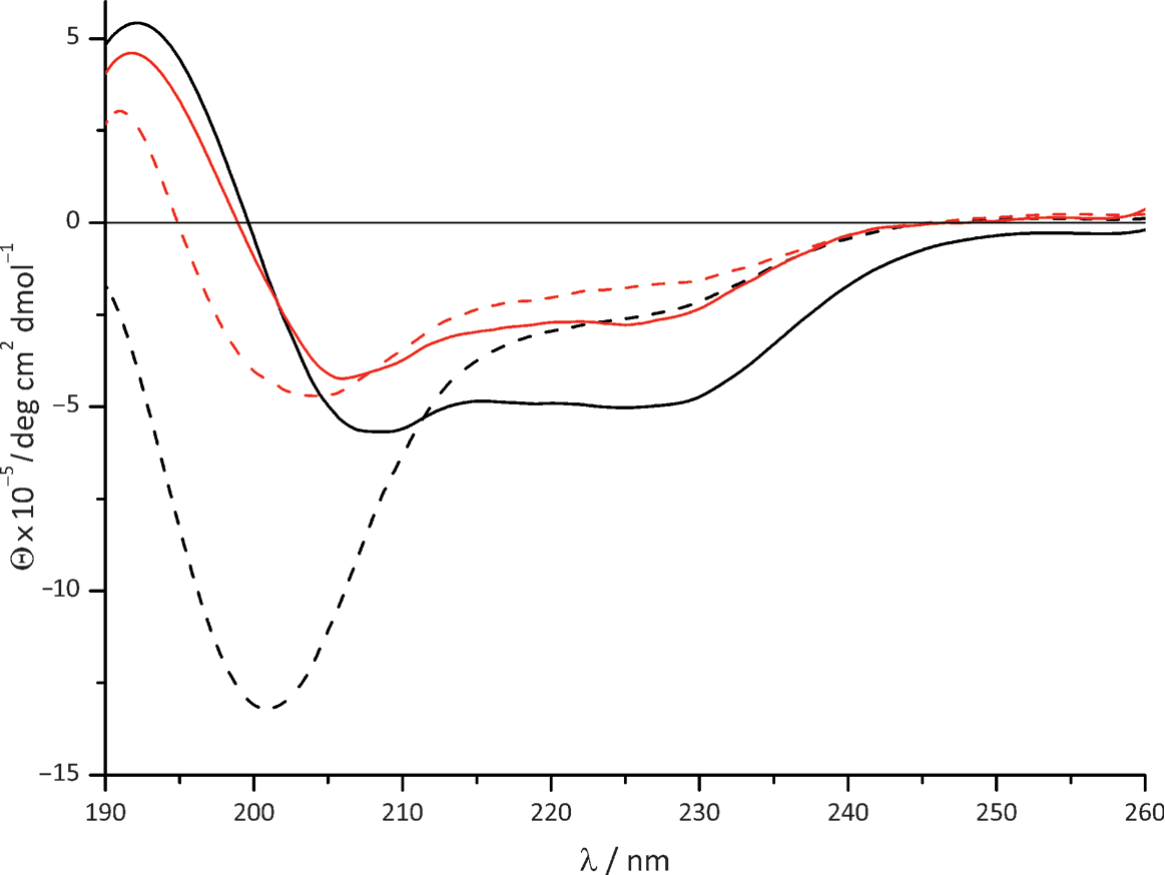
CD spectra of Zf13 IDAO70 (14 μm; black) and Zf13 TACN85 (8.5 μm; red) in the absence (– – –) and presence (—) of 130 μm Zn^2+^.

CD spectra were also recorded for the full-length Zf13 analogues. As indicated for Zf13 TACN85 and Zf13 IDAO70 analogues, the negative maximum at 200 nm undergoes a slightly larger hypsochromic shift of about 6 nm after Zn^2+^ addition. In the case of Zf13 IDAO70, the shift of the negative maximum around 200 nm was accompanied with a strong negative Cotton effect and a shoulder at 222 nm, which is also typical of the zinc-finger fold (Figure 6).^21^

**Figure 7:**
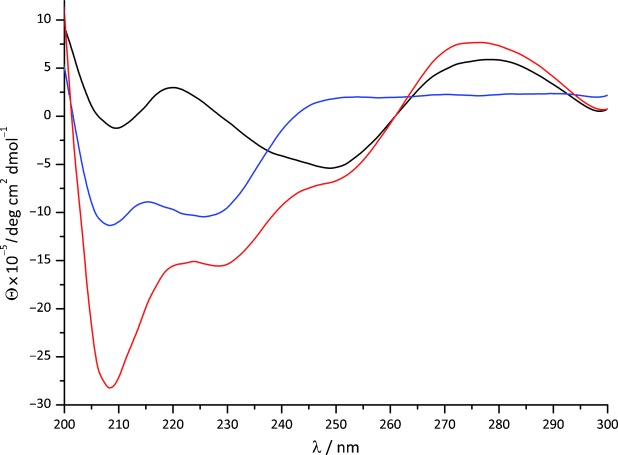
CD spectra of ds DNA (6.5 μm; —), Zf13 IDAO70 (21 μm; —), and the Zf13 IDAO70–ds DNA complex (3 mm; —) in phosphate buffer in the presence of excess zinc (130 μm).

The interaction of zinc-finger constructs with double-stranded (ds) DNA was further investigated by means of CD spectroscopy. The 28-base pair (bp) DNA sequence (^5‘^CTTACGCCCACGCTGATCACACACACAC^3‘^) contains the binding site of Zif268. CD spectra of the ds DNA and Zf13 IDAO70 were recorded separately in phosphate buffer and as a complex in the presence of zinc (Figure 7). The CD spectrum of the Zf13 IDAO70–ds DNA complex shows a significant decrease in the negative maximum at 205 nm and an increased and shifted positive maximum at 278 nm, similar to the spectrum reported previously for DNA binding of the native Zif268.8b, 22 DNA binding of the zinc finger Zf13 IDAO70 seems not to be affected by introduction of an additional metal binding site.

## Conclusion

The semi-synthesis of the natural 90-amino-acid long sequence of the zinc-finger domain of Zif268 is described. Zif268 was modified in the third subunit by a semi-synthetic approach, using expressed protein ligation (EPL) of the chemically synthesized segment with the protein, obtained by recombinant protein expression. Six Zf13 variants were obtained containing additional metal-chelating side chains, a fluorophore, and an acetylene side chain, allowing further postsynthetic modifications by [2+3] cycloaddition. The integrity of the zinc-finger fold was confirmed by CD analysis of the modified zinc fingers. Furthermore, evidence for DNA binding of the full-length construct Zf13 IDAO70 was obtained by CD spectroscopy. Zif268 provides a scaffold for a selective DNA binding peptide that can be subjected to chemical modifications, such as the incorporation of additional metal binding sites or fluorophores without losing its DNA-binding potential.

## Experimental Section

**Materials**: All *N*-α-Fmoc-protected amino acids were purchased from Nova Biochem (Darmstadt, Germany), GL Biochem (Shanghai, China), Fluka (Taufkirchen, Germany), Bachem (Bubendorf, Switzerland), Merck (Darmstadt, Germany), and IRIS Biotech (Marktredwitz, Germany). All other chemicals were purchased from Sigma–Aldrich (Taufkirchen, Germany), ABCR (Karlsruhe, Germany), Acros Organics (Geel, Belgium), Alfa Aesar (Karlsruhe, Germany), New England Biolabs (Ipswich, USA), Macherey&Nagel (Düren, Germany). Preloaded Gly or Lys-*p*-benzyloxybenzyl alcohol resin (Wang resin, 0.3 mmol g^−1^), *N*-hydroxybenzotriazole (HOBt), *O*-benzotriazole-*N*,*N*,*N′*,*N′*-tetramethyl-uronium-hexafluorophosphate (HBTU) were purchased from GL Biochem (Shanghai, China). *N*,*N′*-Diisopropylcarbodiimide (DIC) and *N*-ethyldiisopropylamine (DIPEA) were obtained from IRIS Biotech (Marktredwitz, Germany). 5(6)-Carboxyfluoresceine (CF), 2-mercaptoethane sulfonate sodium (MESNa) and *N*-methyl-2-pyrrolidinon (NMP) were purchased from Fluka (Taufkirchen, Germany). Trifluoroacetic acid (TFA) was bought from Roth (Karlsruhe, Germany).

**ESI-MS**: Data were obtained with a Finnigan instrument (type LCQ) or Bruker spectrometers (types Apex-Q IV 7T, HCT ultra, and micrOTOF API). High-resolution (HR) spectra were obtained with the Bruker Apex-Q IV 7T or the Bruker micrOTOF.

**RP-HPLC**: All RP-HPLC analyses were performed on instruments from GE Healthcare or Jasco. For all semi-preparative/preparative RP-HPLC, we used a Pharmacia Äkta basic device (GE Healthcare) with a gradient of eluent A (0.1 % TFA in H_2_O) to eluent B (0.1 % TFA in MeCN/H_2_O 8:2) with a flow rate of 3 mL min^−1^ (semipreparative)/10 mL min^−1^ (preparative) were performed. Preparative purification was performed on a Phenomenex column Jupiter, RP-C18, 250×20 mm, 5 μm, 80 Å. For semi-preparative purification, a Phenomenex column Jupiter, RP-C18, 250×10 mm, 5 μm, 80 Å was used. For analytical RP-HPLC, a semi-micro-HPLC system from Jasco was used, applying a gradient of eluent A (0.1 % TFA in H_2_O) to eluent B (0.1 % TFA in MeCN) with a flow rate of 1 mL min^−1^. In these cases, a Phenomenex column RP-C18, 250×4.6 mm, 5 μm was used. UV detection was performed at 215 nm, 254 nm and, for the fluorophore-labeled peptides, at 444 nm.

**Circular dichroism (CD) spectroscopy**: CD spectra were recorded on a Jasco-810 spectropolarimeter equipped with a Jasco PTC432S temperature controller. Prior to usage, the sample cell was flushed with nitrogen. For CD spectra recording zinc-finger derivatives, 1 mm quartz glass precision cells were used. The peptide concentrations were adjusted to between 5–30 μm for all derivatives. The spectra were recorded at 20 °C in a wavelength range of 260–190 nm for the zinc fingers and from 300–200 nm for the DNA binding studies with 1.0 nm bandwidth in continuous mode, 1.0 s response and a scan speed of 100 nm min^−1^. Eight spectra were averaged. Spectra were background-corrected, smoothed (Savitzky–Golay), and depicted as molar ellipticity (Θ ×10^−5^ deg cm^2^ dmol^−1^).

**Cloning of Zf12**: The cDNA encoding the murine transcription factor Zif268 (EgrI) was purchased from ATCC (Wesel, Germany). After amplifying the desired DNA fragment of the two zinc-finger domains (632–827) by polymerase chain reaction (PCR) using the forward ^5‘^GGTGGTGCTAGCGAACGCCCATATGCTTGCCCTG^3’^ and reverse ^5‘^GGTGGTTGCTCTTCCGCAGTCCTTCTGTCTTAAATGGATTTTG^3’^ primers from Sigma–Aldrich (Taufkirchen, Germany), and purification by agarose-gel electrophoresis, the fragment was ligated into the cloning vector pGEM-T from Promega (Mannheim, Germany). After digestion of the cloning vector pGEM-T and expression vector pTXB1 from New England Biolabs (Ipswich, USA), using the restriction enzymes SapI and NheI, the DNA insert and vector were subsequently ligated. To confirm an in-frame cloning of Zf12 (**1**), DNA sequencing was performed.

**Protein expression of Zf12 in** ***E. coli***: *E. coli* ER2566 cells were transformed with the pTXB1/Zf12 (632–827) DNA plasmid and grown in lysogeny broth (LB) or 2*YT medium containing carbenicilline (100 μg mL^−1^). A preparatory culture (5 mL) was used to inoculate the expression culture. The cells were cultivated at 200 rpm at 37 °C, until the culture reached an optical density at 600 nm (*OD*_600_) of 0.6–0.8. The culture was then induced by adding 0.4 mm isopropyl β-D-1-thiogalactopyranoside (IPTG) and incubated again overnight at 16 °C. The cells were harvested by centrifugation at 4 °C at 9000 *g* and lysed in buffer A: 2-[4-(2-hydroxyethyl)piperazin-1-yl]ethanesulfonic (HEPES) buffer (20 mm), NaCl (500 mm), tris(2-carboxyethyl)phosphine (0.1 mm), Tween20 (0.1 %), pH 8, and 20 μm phenylmethylsulfonylfluoride (PMSF) was added before incubating on ice for 30 min. Complete lysis was accomplished by pulsed sonication (5×45 s). The soluble extracts of the protein were isolated by centrifugation at 23 000 *g* for 20 min at 4 °C. Purification and isolation of Zf12 (**1**) was enabled by an attached chitin binding domain (CBD) as follows: a column (GE Healthcare, XK 26/20) filled with 100 mL chitin beads (New England Biolabs) was equilibrated at 4 °C with water (2×bed volumes) and buffer A (10×bed volumes). Loading of the cell lysate proceeded overnight at a flow rate of 1 mL min^−1^ (10 times). Unspecifically bound *E. coli* proteins were removed by washing with buffer A (3×bed volumes). The intein fusion protein of Zf12 was cleaved in the presence of buffer B: HEPES (20 mm), MESNa (250 mm, pH 6) over 70 h at 4 °C. The peptide thioester was eluted with buffer A. The complete procedure was monitored at 4 °C by UV absorption (280 nm, Äkta Prime Plus, GE Healthcare). Analysis of the peptide thioester was carried out by SDS-PAGE (tris-tricine 10–20 %), RP-HPLC on a C18 semi-preparative column (Phenomenex column Jupiter RP-C18, 250×10 mm, 5 μm, 80 Å) with 0.1 % TFA in H_2_O (A) and 0.1 % TFA in H_2_O/MeCN (20:80) (B) as eluting system with a 20→50 % gradient of B over 30 min at a flow rate of 3 mL min^−1^, and HRMS-ESI.

**Reduced Zf12 (1)**: H_2_N-ASERPYACPVESCDRRFSRSDELTRHIRIHTGQKPFQCRICMRNFSRSDHLTTHIRTHTGEKPFA—OH; *M*_r_=7794.8 (C_328_H_520_N_110_O_98_S_7_); MS (ESI): *m*/*z*=650.57 [*M*+12H]^12+^, 709.62 [*M*+11H]^11+^, 780.48 [*M*+10H]^10+^; HRMS-ESI: *m/z* calcd for C_328_H_520_N_110_O_98_S_7_: 709.3348 [*M*+11H]^11+^, found: 709.3460; 780.1786 [*M*+10H]^10+^, found: 780.1788.

**SPPS of Zf3 and respective modifications**: The peptides were synthesized by standard Fmoc/*tert*-butyl peptide synthesis on solid support on preloaded Wang resin, 0.3 mmol g^−1^ using a microwave-supported automated peptide synthesizer LibertyTM (CEM Cooperation, Matthews, NC, USA). The side chain protection groups were *tert*-butyl for aspartic acid, glutamic acid, serine, tyrosine; *tert*-butyloxycarbonyl (Boc) or allyloxycarbonyl (alloc) for lysine; triphenylmethyl (trityl) for cysteine, glutamine and histidine; and 2,2,4,6,7-pentamethyl-dihydrofurane-5-sulfonyl (PBF) for arginine. Resins were swollen in CH_2_Cl_2_ for 30 min. The coupling protocol was adjusted to the different needs of the amino acids coupled. Fmoc amino acids were used as 0.2 m solutions in *N*-methylpyrrolidone (NMP). Coupling was performed with 0.5 m HBTU/HOBt (5 equiv) in DMF, 0.2 m amino acids in NMP (5 equiv) and 2 m DIPEA (10 equiv) in NMP. Double coupling of the amino acids arginine, cysteine and aspartic acid was performed to enhance the coupling efficiency. Capping was performed after every coupling cycle using a solution of acetic anhydride (10 %), DIPEA (5 %), and HOBt (0.2 %) in NMP. All reaction steps were performed under microwave conditions (20 W, 300 s at 75 °C) and N_2_ mixing. Arginine was coupled under N_2_ mixing (1500 s) at RT followed by microwave-assisted coupling (300 s) at 75 °C. To minimize racemization during coupling, cysteine derivatives were coupled for 300 s at 50 °C. Histidine was coupled without using the microwave supply for 60 min.^23^ The Fmoc-protecting group was removed by two deprotection cycles with 20 % piperidine in NMP (30 s, 180 s). Final deprotection of the Fmoc group was performed for all peptides except the orthogonal Lys(alloc)-protected derivatives required for further fluorophore coupling. Prior to cleavage, the resins were washed with DMF/CH_2_Cl_2_ (1:1, 5×5 mL), CH_2_Cl_2_ (3×5 mL), EtOH (3×5 mL), and CH_2_Cl_2_ (5×5 mL) and dried overnight. Cleavage from the resin and simultaneous removal of all side chain protecting groups was performed separately for all derivatives in a 10 mL syringe (Becton, Dickinson & Co., Franklin Lakes, NJ, USA) by adding TFA/H_2_O/triethylsilyl/1,2-ethanedithiol (*v*/*v*/*v*/*v*=95:2:1:2, 10 mL g^−1^ resin) and shaking for 2 h at RT. The peptides were precipitated from ice-cold *tert*-butylmethylether, isolated by centrifugation (9000 rpm, 4 °C) and washed with ice-cold Et_2_O (3×); the pellet was subsequently lyophilized from H_2_O using a freeze dryer (Martin Christ GmbH, Osterode am Harz, Germany). The lyophilized peptides were purified by semi-preparative/preparative RP-HPLC on C18 columns (Phenomenex column Jupiter RP-C18, 250×10 mm, 5 μm, 80 Å with 0.1 % TFA in H_2_O (A) and 0.1 % TFA in H_2_O/MeCN (20:80) (B) as eluting system with a 20–50 % gradient of B over 30 min at a flow rate of 3/10 mL min^−1^ and lyophilized again. The peptides were analyzed by HRMS-ESI.

**Introduction of modifications**: All modifications were accessible within the SPPS protocol. In the case of the attachment of the fluorophore instead of Fmoc–Cys(Trt)—OH the complementary Boc derivative was coupled as N-terminal amino acid. 5(6)-Carboxyfluoresceine was introduced by standard coupling to the side chain of Lys 89, which was orthogonally protected by an alloc group. Prior to cleavage from the resin, the alloc group was removed by treatment with MeNHBH_3_ (30 equiv) and [Pd(PPh_3_)_4_] (0.1 equiv) over 4 h in DMF at RT. 5(6)-Carboxyfluoresceine was activated with HOBt and DIC, and coupled to the lysine side chain over 2 h at RT. Arg 70 was exchanged for the synthesized artificial amino acid Fmoc–Pra—OH to allow Click chemistry and also for the artificial metal-chelating amino acid Fmoc–IDAO—OH, which was coupled manually using HOAt/HBTU coupling reagents. His 85 was exchanged by the metal-chelating amino acid Fmoc–TACN—OH.^12^

**Analysis of Zf3 and its modifications**: The purified reduced peptides were analyzed by RP-HPLC on C18 semi-preparative column (Phenomenex column RP-C18, 250×10 mm, 5 μm), with 0.1 % TFA in H_2_O (A) and 0.1 % TFA in H_2_O/MeCN (20:80) (B) as eluting system at a flow rate of 3 mL min^−1^, and HRMS-ESI.

**Zf3**: H_2_N—CDICGRKFARSDERKRHTKIHLRQKG—OH; *M*_r_=3137.69 (C_131_H_222_N_50_O_36_S_2_); RP-HPLC: gradient B: 20→50 % over 30 min, *t*_R_=12.0 min; MS (ESI): *m/z*=785.4 [*M*+4H]^4+^, 1046.7 [*M*+3H]^3+^; HRMS-ESI: *m/z* calcd for C_131_H_222_H_50_O_36_S_2_: 523.7830 [*M*+4H]^4+^, found: 523.7832; calcd: 628.3382 [*M*+5H]^5+^, found: 628.3388; calcd: 785.1709 [*M*+4H]^4+^, found: 785.1712.

**Zf3 A85 (2)**: H_2_N—CDICGRKFARSDERKRHTKIALRQKDG—OH; *M*_r_=3188.71 (C_132_H_225_N_49_O_39_S_2_); RP-HPLC: gradient B: 20→50 % over 30 min, *t*_R_=12.76 min; MS (ESI): *m/z*=532.12 [*M*+6H]^6+^, 638.14 [*M*+5H]^5+^, 797.42 [*M*+4H]^4+^, 1063.23 [*M*+3H]^3+^; HRMS-ESI: *m/z* calcd for C_132_H_225_H_59_O_39_S_2_: 638.1392 [*M*+5H]^5+^, found: 638.1391.

**Zf3 A85 l89 (3)**: H_2_N—CDICGRKFARSDERKRHTKIALRQK(FL)DG—OH; *M*_r_=3547.02 (C_153_H_235_N_49_O_45_S_2_); RP-HPLC: gradient B: 20→50 % over 30 min, *t*_R_=19.83 min; MS (ESI): *m/z*=591.63 [*M*+6H]^6+^, 709.75 [*M*+5H]^5+^, 886.94 [*M*+4H]^4+^, 1182.25 [*M*+3H]^3+^; HRMS-ESI: *m/z* calcd for C_153_H_235_H_49_O_45_S_2_: 709.9493 [*M*+5H]^5+^, found: 709.9492; calcd: 886.6835 [*M*+4H]^4+^, found: 886.6832.

**Zf3 Pra70 (4)**: H_2_N—CDICGPRAKFARSDERKRHTKIHLRQKDG—OH; *M*_r_=3193.69 (C_134_H_220_N_48_O_39_S_2_); RP-HPLC: gradient B: 20→50 % over 30 min, *t*_R_=10.37 min; MS (ESI): *m/z*=532.95 [*M*+6H]^6+^, 639.34 [*M*+5H]^5+^, 798.92 [*M*+4H]^4+^, 1064.88 [*M*+3H]^3+^; HRMS-ESI: *m/z* calcd for C_134_H_220_H_48_O_39_S_2_: 639.1308 [*M*+5H]^5+^, found: 639.1309; calcd: 798.6617 [*M*+4H]^4+^, found: 798.6614.

**Zf3 TACN85 (5)**: H_2_N—CDICGRKFARSDERKRHTKITACNLRQK—OH; *M*_r_=3234.8 (C_136_H_235_N_53_O_35_S_2_); RP-HPLC: gradient B: 5→40 % over 30 min, *t*_R_=21.85 min; MS (ESI): *m*/*z*=648.36 [*M*+5H]^5+^, 809.95 [*M*+4H]^4+^, 1079.27 [*M*+3H]^3+^.

**Zf3 IDAO70 (6)**: H_2_N—CDICGIDAOKFARSDERKRHTKIHLRQK—OH; *M*_r_=3154.67; RP-HPLC: gradient B: 20→50 % over 30 min, *t*_R_=13.5 min; MS (ESI): *m/z*=631.94 [*M*+5H]^5+^, 789.67 [*M*+4H]^4+^, 1052.55 [*M*+3H]^3+^; HRMS-ESI: *m/z* calcd for C_132_H_219_H_47_O_39_S_2_: 631.5312 [*M*+5H]^5+^, found: 631.5316; calcd: 789.1622 [*M*+4H]^4+^, found: 789.1618.

**Ligation of Zf13 and its modifications**: The N-terminal Cys 65 of the different Zf3 peptides had to be available for nucleophilic attack at the thioester of **1** during the ligation step. Therefore, the disulfide bridges in Zf3 and the other constructs were first reduced. The peptide (3 mm) was dissolved in phosphate buffer (10 mm, pH 4, TCEP 20 mm) and stirred overnight at RT under inert conditions. Complete reduction of the peptide was confirmed by ESI-MS. Subsequently, peptide thioester **1** (1 mm) was added in phosphate buffer (500 mm, pH 8) and the pH of the solution was adjusted to pH 7.8–8. As determined by RP-HPLC, the conversion of the two peptides into the ligation product was observed after stirring for 6–12 h at RT under inert conditions.

**Analysis of the ligation products**: The ligation products were analyzed by RP-HPLC on an analytical C18 column (Phenomenex column RP-C18, 250×4.6 mm, 5 μm) with 0.1 % TFA in H_2_O (A) and 0.1 % TFA in MeCN (B) as eluting system with a 20–50 gradient of B over 30 min at a flow rate of 1 mL min^−1^ and ESI-MS.

**Zf13**: H_2_N—ASERPYACPVESCDRRFSRSDELTRHIRIHTGQKPFQCRICMRNFSRSDHLTTHIRTHTGEKPFACDICGRKFARSDERKRHTKIHLRQKG—OH; *M*_r_=10 794.4 (C_457_H_732_N_160_O_131_S_7_); RP-HPLC: gradient B: 20→50 in 30 min, *t*_R_=18.1 min; ESI-MS: *m/z*=721.3 [*M*+15H]^15+^, 771.9 [*M*+14H]^14+^, 830.9 [*M*+13H]^13+^, 900.1 [*M*+12H]^12+^, 981.7 [*M*+11H]^11+^, 1080.0 [*M*+10H]^10+^.

**Zf13 A85**: H_2_N—ASERPYACPVESCDRRFSRSDELTRHIRIHTGQKPFQCRICMRNFSRSDHLTTHIRTHTGEKPFACDICGRKFARSDERKRHTKIALRQKDG—OH; *M*_r_=10 843.4 (C_458_H_741_N_159_O_134_S_7_); RP-HPLC: gradient B 20→40 % over 40 min, flow rate=0.3 mL min^−1^, *t*_R_=27.5 min; MS (ESI): *m/z*=603.3 [*M*+18H]^18+^, 678.4 [*M*+16H]^16+^, 723.5 [*M*+15H]^15+^, 834.8 [*M*+13H]^13+^, 904.4 [*M*+12H]^12+^, 986.7 [*M*+11H]^11+^, 1085.2 [*M*+10H]^10+^, 1205.7 [*M*+9H]^9+^, 1401.1 [*M*+8H]^8+^, 1356.3 [*M*+7H]^7+^; HRMS: *m/z* calcd for C_458_H_741_N_159_O_134_S_7_: 678.3456 [*M*+16H]^16+^, found: 678.3536; calcd: 1085.248 [*M*+10H]^10+^, found: 1085.237; calcd: 1205.720 [*M*+9H]^9+^, found: 1205.703; calcd: 1356.309 [*M*+7H]^7+^, found: 1356.291.

**ZF13 A85 L89**: H_2_N—ASERPYACPVESCDRRFSRSDELTRHIRIHTGQKPFQCRICMRNFSRSDHLTTHIRTHTGEKPFACDICGRKFARSDERKRHTKIALRQK(FL)DG—OH; *M*_r_=11 215.5 (C_480_H_753_N_159_O_140_S_7_); RP-HPLC: gradient B: 20→50 % over 30 min, *t*_R_=18.62 min; MS (ESI): *m/z*=701.3 [*M*+16H]^16+^, 747.9 [*M*+15H]^15+^, 801.3 [*M*+14H]^14+^, 862.8 [*M*+13H]^13+^, 934.6 [*M*+12H]^12+^, 1019.5 [*M*+11H]^11+^, 1121.2 [*M*+10H]^10+^, 1245.6 [*M*+9H]^9+^, 1401.1 [*M*+8H]^8+^, 1601.1 [*M*+7H]^7+^, 1867.6 [*M*+6H]^6+^.

**ZF13 PRA70**: H_2_N—ASERPYACPVESCDRRFSRSDELTRHIRIHTGQKPFQCRICMRNFSRSDHLTTHIRTHTGEKPFACDICGPRAKFARSDERKRHTKIHLRQKDG—OH; *M*_r_=10 848.4 (C_460_H_736_N_158_O_134_S_7_); RP-HPLC: gradient B: 27→50 % over 30 min, *t*_R_=10.56 min; MS (ESI): *m/z*=640.1 [*M*+17H]^17+^, 679.8 [*M*+16H]^16+^, 725.0 [*M*+15H]^15+^, 776.8 [*M*+14H]^14+^, 836.3 [*M*+13H]^13+^, 905.5 [*M*+12H]^12+^, 987.3 [*M*+11H]^11+^, 1085.9 [*M*+10H]^10+^.

**ZF13 TACN85**: H_2_N—ASERPYACPVESCDRRFSRSDELTRHIRIHTGQKPFQCRICMRNFSRSDHLTTHIRTHTGEKPFACDICGRKFARSDERKRHTKITACNLRQK—OH; *M*_r_=10 893.5 (C_462_H_751_N_163_O_130_S_7_); RP-HPLC: gradient B: 20→50 % over 30 min, *t*_R_=18.9 min; MS (ESI): *m/z*=574.5 [*M*+19H]^19+^, 606.4 [*M*+18H]^18+^, 642.0 [*M*+17H]^17+^, 682.0 [*M*+16H]^16+^, 727.4 [*M*+15H]^15+^, 779.3 [*M*+14H]^14+^, 891.1 [*M*+13H]^13+^, 908.9 [*M*+12H]^12+^, 991.4 [*M*+11H]^11+^, 1090.4 [*M*+10H]^10+^.

**ZF13 IDAO70**: H_2_N—ASERPYACPVESCDRRFSRSDELTRHIRIHTGQKPFQCRICMRNFSRSDHLTTHIRTHTGEKPFACDICGIDAOKFARSDERKRHTKIHLRQK—OH; *M*_r_=10 811.4 (C_458_H_737_N_157_O_134_S_7_); RP-HPLC: gradient B: 20→50 % over 30 min, *t*_R_=18.74 min; MS (ESI): *m/z*=570.2 [*M*+19H]^19+^, 601.8 [*M*+18H]^18+^, 637.1 [*M*+17H]^17+^, 676.9 [*M*+16H]^16+^, 721.9 [*M*+15H]^15+^, 773.4 [*M*+14H]^14+^, 832.8 [*M*+13H]^13+^, 902.1 [*M*+12H]^12+^, 983.9 [*M*+11H]^11+^, 1082.2 [*M*+10H]^10+^.

## References

[1a] Klug A (1999). J. Mol. Biol.

[1b] von Hippel PH, McGhee JD (1972). Annu. Rev. Biochem.

[1c] Mitchell PJ, Tijan R (1989). Science.

[1d] McGhee JD, von Hippel PH (1974). J. Mol. Biol.

[2] Rubin GM, Yandell MD, Wortman JR, Gabor GL, Miklos, Nelson CR, Hariharan IK, Fortini ME, Li PW, Apweiler R, Fleischmann W, Cherry JM, Henikoff S, Skupski MP, Misra S, Ashburner M, Birney E, Boguski MS, Brody T, Brokstein P (2000). Science.

[3] Venter JC, Adams MD, Myers EW, Li PW, Mural RJ, Sutton GG, Smith HO, Yandell Mark, Evans CA, Holt RA, Gocayne JD, Amanatides Peter, Ballew RM, Huson DH, Wortman JenniferRusso, Zhang Qing, Kodira CD, Zheng XH, Chen Lin, Skupski Marian (2001). Science.

[4a] Arakawa H, Nagase H, Ogawa M, Nagata M, Fujiwara T, Takahashi E, Shin S, Nakamura Y (1995). Cytogenet. Cell Genet.

[4b] Miller J, McLachlan AD, Klug A (1985). EMBO J.

[5a] Krishna SS (2003). Nucleic Acids Res.

[5b] Iuchi S (2001). Cell. Mol. Life Sci.

[6] Elrod-Erickson M, Rould MA, Nekludova L, Pabo CO (1996). Structure.

[7] Anzellotti AI, Farrell NP (2008). Chem. Soc. Rev.

[8a] Pavletich NP, Pabo CO (1991). Science.

[8b] Elrod-Erickson M, Pabo CO (1999). J. Biol. Chem.

[9a] Matthews J, Loughlin F, Mackay J (2008). Curr. Opin. Struct. Biol.

[9b] Nadler A, Hain C, Diederichsen U (2009). Eur. J. Org. Chem.

[10] Lipscomb WN, Sträter N (1996). Chem. Rev.

[11a] Negi S, Imanishi M, Matsumoto M, Sugiura Y (2008). Chem. Eur. J.

[11b] Waldron KJ, Rutherford JC, Ford D, Robinson NJ (2009). Nature.

[11c] Lu Y, Yeung N, Sieracki N, Marshall NM (2009). Nature.

[12a] Guillena G, Rodríguez G, van Koten G (2002). Tetrahedron Lett.

[12b] Johnsson K, Allemann RK, Widmer H, Benner SA (1993). Nature.

[13a] Harris KL, Lim S, Franklin SJ (2006). Inorg. Chem.

[13b] Hellinga HW (1996). Curr. Opin. Struct. Biotech.

[13c] Lu Y, Berry SM, Pfister TD (2001). Chem. Rev.

[13d] DeGrado W, Summa C, Pavone V, Nastri F, Lombardi A (1999). Annu. Rev. Biochem.

[15] Schägger H (2006). Nat. Protoc.

[16a] Nomura A, Sugiura Y (2002). Inorg. Chem.

[16b] Nomura A, Sugiura Y (2004). Inorg. Chem.

[16c] Simpson RJ, Cram ED, Czolij R, Matthews JM, Crossley M, Mackay JP (2003). J. Biol. Chem.

[17a] Jares-Erijman EA, Jovin TM (2003). Nat. Biotechnol.

[17b] Giannetti A, Citti L, Domenici C, Tedeschi L, Baldini F, Wabuyele MB, Vo-Dinh T (2006). Sens. Actuators B.

[18a] Tornøe CW, Christensen CC, Meldal M (2002). J. Org. Chem.

[18b] Kolb HC, Finn MG, Sharpless KB Angew. Chem.

[18c] Mindt TL, Struthers H, Brans L, Anguelov T, Schweinsberg C, Maes V, Tourwe D, Schibli R (2006). J. Am. Chem. Soc.

[19] Gajewski M, Seaver B, Esslinger CS (2007). Bioorg. Med. Chem. Lett.

[20] Dawson PE, Muir TW, Lewis IC, Kent SB (1994). Science.

[21a] Frankel AD, Berg JM, Pabo CO (1987). Proc. Natl. Acad. Sci. USA.

[21b] Negi S, Itazu M, Imanishi M, Nomura A, Sugiura Y (2004). Biochem. Biophys. Res. Commun.

[21c] Kopera E, Schwerdtle T, Hartwig A, Bal W (2004). Chem. Res. Toxicol.

[22] Beligere GS, Dawson PE (1999). Biopolymers.

[23] Bacsa B, Bösze S, Kappe CO (2010). J. Org. Chem.

